# Pain

**Published:** 1894-06

**Authors:** 


PAIN without fever, says a prominent physician, may be very severe, and may cause much suffering, but in acute attacks it is not dangerous. "If you had this amount of pain that you complain of," he said to a patient who had hastily summoned him, "in any inflammatory disease, you would be in a raging fever; if you have no fever you need never worry." Most serious illnesses are preceded by a chill. This is a symptom which should never be disregarded, and it is always safest to put a child to bed and stop its food. Warmth and dieting will be found to be the best remedy for any ordinary indisposition, while for the beginning of any serious trouble it is often the only thing that can be done until the disease declares itself.—*Good Health.*

